# Comparison of a frozen human foreskin fibroblast cell assay to an enzyme immunoassay and toxigenic culture for the detection of toxigenic *Clostridium difficile*^☆^^☆☆^^★^

**DOI:** 10.1016/j.diagmicrobio.2012.09.013

**Published:** 2013-01

**Authors:** Alastair J. Strachan, Natalie E. Evans, O. Martin Williams, Robert C. Spencer, Rosemary Greenwood, Chris J. Probert

**Affiliations:** a Department of Clinical Sciences, University of Bristol, Bristol, UK; b Health Protection Agency Microbiology Services, Bristol, Bristol, UK; c Research Design Service–South West, University Hospital Bristol NHS Foundation Trust, Bristol, UK; d Department of Gastroenterology, University of Liverpool, Liverpool, UK

**Keywords:** *Clostridium difficile*, Enzyme immunoassay, Toxigenic culture, Cytotoxin

## Abstract

This study set out to validate the Hs27 ReadyCell assay (RCCNA) as an alternative CCNA method compared against a commonly used commercial enzyme immunoassay (EIA) method and toxigenic culture (TC) reference standard. A total of 860 samples were identified from those submitted to the Health Protection Agency microbiology laboratories over a 30-week period. RCCNA performed much better than EIA when using TC as a gold standard, with sensitivities of 90.8% versus 78.6% and positive predictive value of 87.3% to 81.9%, respectively. The Hs27 Human Foreskin Fibroblast ReadyCells are an easy-to-use and a sensitive CCNA method for the detection of toxigenic *Clostridium difficile* directly from stool. A turnaround time of up to 48 h for a negative result and possible need for repeat testing make it an unsuitable method to be used in most clinical laboratory setting.

## 1 Introduction

*Clostridium difficile* is the most common cause of nosocomial colitis, with symptoms ranging from asymptomatic carriage to severe diarrhoea, pseudomembranous colitis, toxic megacolon, and death (Williams and Spencer, 2009). These symptoms are the result of the toxins excreted by the bacteria; non–toxin-producing strains of the bacteria are nonpathogenic (Williams and Spencer, 2009). A fast and accurate method for the diagnosis of the infection is required to improve patient care and reduce the risk of transmission. Since 2007, the prevalence of *C. difficile* infection (CDI) has decreased in the UK (Health Protection Agency, 2011), although it is still rising in other countries (Crobach et al., 2009).

The choice of laboratory test can have a significant impact on the accuracy of a *C. difficile* diagnosis (Crobach et al., 2009; Carroll, 2011; Planche and Wilcox, 2011). Cell cytotoxin neutralisation assays (CCNA) are recommended as the gold standard (GS) for detecting *C. difficile* toxin B in a laboratory environment (Crobach et al., 2009; Carroll, 2011; Planche and Wilcox, 2011), but the drawbacks of using this method including the 48-h turnaround time, cell line maintenance, and technical expertise have led to many laboratories choosing enzyme immunoassays (EIA) as their diagnostic method; EIA have a shorter turnaround times and cost less than CCNA. EIAs are commonly used to detect toxins A and B, but it has been reported that their ability to accurately diagnose a toxigenic *C. difficile* infection can be substandard (Carroll, 2011).

A new commercial method of cytotoxin testing using Hs27 Human Foreskin Fibroblast (HFF) ReadyCells (Diagnostic Hybrids, Athens, OH, USA) and requiring no cell line maintenance was recently introduced to overcome the problems of the EIA and previous CCNA testing methods. These cells are an alternative to the commonly used Vero cells whose performance has been well documented (Eastwood et al., 2009; Novak Weekley et al., 2010). Although the merits of CCNA testing for *C. difficile* diagnosis are also known, there is little published experience of the new method. A recent review highlighted the availability of commercially available frozen HFF cells but noted their use requires validation (Planche and Wilcox, 2011).

The aim of this study was to assess Hs27 ReadyCell assay (RCCNA) as an alternative CCNA method and to compare their diagnostic capability for toxigenic *C. difficile* against a commonly used commercial EIA method and toxigenic culture (TC) reference standard.

## 2 Materials and methods

Routine clinical samples sent to the laboratory were tested for *C. difficile* if they matched stool form types 5 to 7 on the Bristol Stool Scale (Lewis and Heaton, 1997) and met any of the following patient criteria: aged ≥65 years, taking or had recently taken antibiotics, a hospital inpatient, immunosuppressed, requested by the patient's clinician. From those who met these criteria, samples were selected that were fresh (<24 h since being collected), >5 mL in volume, from patients aged ≥18 years old who had diarrhoea for >24 h.

### 2.1 Enzyme immunoassay

The Premier *C. difficile* Toxin A & B microwell EIA (Meridian Bioscience, Cincinnati, OH, USA) was used in accordance with the Health Protection Agency (HPA) standard operating procedures on the DS2 analyser (Launch Diagnostics, Kent, UK) by HPA staff. Optical densities (OD) were determined using the manufacturer's guidelines at 450 and 630 nm; a positive result was determined by an OD ≥0.1 and a negative result by an OD <0.1.

### 2.2 Cell cytotoxin neutralization assay

Human foreskin fibroblast Hs27 ReadyCells (Diagnostic Hybrids) were used for the CCNA. One millilitre of stool was frozen on receipt and testing performed in batches. Samples were defrosted and added to 3 mL of specimen diluent (dilution 1:4) and centrifuged at 3500 × *g* for 10 min. The supernatant was removed and passed through a 0.45-micron sterile syringe filter (Whatman, Dassel, Germany). Two sterile 1.5-mL Eppendorf tubes were prepared for each sample, 1 containing 0.2 mL of specimen diluent, the other 0.2 mL of antitoxin control, with 0.2 mL specimen filtrate added to both (dilution 1:8) and left to incubate at room temperature for 30 min. The HFF ReadyCells were removed from storage at −70 °C and defrosted in the ReadyCell heat block (Diagnostic Hybrids) for 4 min. The cells' maintenance media was removed and 0.8 mL of fresh Refeed Medium (Diagnostic Hybrids, Athens, OH, USA) added to each cell vial. With a sterile pipette tip, 0.2 mL of the specimen filtrate and 0.2 mL of the antitoxin control solution were added to separate RCCNA vials. All vials were incubated at 37 °C with 5% CO_2_ for a maximum of 48 h. Cell lines were examined at 24 and 48 h of incubation using an inverted microscope, ×10 magnification, for signs of cytopathic effect (CPE). A positive result was defined by ≥50% cell lysis with no evidence of cytotoxicity in the relevant antitoxin control vial.

#### 2.2.1 Repeat samples

Samples where no clear result could be determined (both specimen and control vial displaying CPE, destruction of cell monolayer) were repeated with titrations of 1:8, 1:16, and 1:32 added to separate RCCNA vials and incubated as above.

### 2.3 Toxigenic culture

All stool samples were processed for TC according to a protocol modified from the one set out by Eastwood et al. (2009). 0.5 mL of stool was added to 0.5 mL of industrial methylated spirit or at a ratio of 1:1 and left to ‘shock’ at room temperature for 30 min. One loop full of the shock was inoculated onto Brazier's agar (Oxoid, Cambridge, UK) and incubated in anaerobic conditions for 48 h. Suspected *C. difficile* colonies were inoculated onto fastidious anaerobic agar (Oxoid) and reincubated for a further 48 h. Positive *C. difficile* culture was determined by meeting all the following: yellow/green colonies under UV fluorescence, a positive latex agglutination (Microgen Bioproducts, Camberley, UK), and the characteristic horse barn odour. Positive *C. difficile* cultures were run on the DS2 analyser for CDT EIA testing as outlined above.

### 2.4 Statistical analysis

Statistical analysis was performed using the IBM SPSS 19 software package (SPSS, Chicago, IL, USA) to provide kappa values and 95% confidence intervals. Sensitivities, specificities, positive predictive values (PPVs), and negative predictive values (NPVs) were also derived for each test method.

## 3 Results

A total of 860 samples were identified from those submitted to the HPA microbiology laboratories from 2 hospital trusts (University Hospital Bristol NHS Foundation Trust and Royal United Hospital Bath) over a 30-week period. The prevalence of *C. difficile* amongst these cases of diarrhoea was 10.9% with EIA, 11.4% with TC, and 11.9% with CCNA.

The results for comparing the 3 toxigenic CDI diagnosis methods are presented in Table 1. The EIA and RCCNA were compared against one another using the TC as the gold standard (GS) reference method to compare and evaluate their results. RCCNA performed much better than EIA when using TC as a GS, with sensitivities of 90.8% (CI 83.3–95.7) and 78.6% (CI 69.1–81.2), respectively. There was only a slight difference in specificity with 98.3% (CI 97.1–99.0) for the RCCNA, only slightly higher than 97.8% (CI 96.5–98.7). RCCNA was better at identifying a true-negative result with a NPV of 98.8% (CI 97.8–99.5). The false-negative rate was 9.2% (4.3–16.7) for RCCNA, compared to a much higher 21.4% [13.8-30.9] for EIA. The EIA false-positive rate of 2.2% (1.3–3.5) was only 0.5% higher than the recorded level of 1.7% (0.9–2.9) for the RCCNA. PPVs were higher for the RCCNA (87.3% [79.2–93.0]) than for the EIA (81.9% [72.6–89.0]).

Kappa values for EIA and RCCNA were 0.777 and 0.876, respectively, showing a higher level of repeatability for the RCCNA when TC is used as GS. When the reliability of each method was evaluated, no statistical significance could be found between EIA and TC (*P* = 0.627), EIA and RCCNA (0.200), and RCCNA against TC (0.523).

A total of 3.3% (28/860) of RCCNA samples included in the study were repeated. Eight-two percent (23/28) were repeated due to ‘bunching’ of the cell monolayer, and a further 18% (5/28) showed CPE in both vials. Samples which needed repeating incurred a further cost of approx £27.00 per sample.

Fifteen percent of positive *C. difficile* cultures were negative on the EIA component of the TC algorithm and therefore resulted as negative for TC. It is possible that these cultures contained nontoxigenic *C. difficile* strains or that the Toxin A & B EIA result was a false negative.

## 4 Discussion

There is still much debate regarding the best method for the diagnosis of toxigenic *C. difficile*, with the need for an accurate and relevant diagnosis often compromised by the requirement of a rapid result. A key advantage of the CCNA test method has been its sensitivity to detect small quantities (1 pg) of *C. difficile* toxin (Lyerly et al., 1988). Due to factors such as reductions in available technical expertise, costs, and cell line maintenance, CCNA methods have not been adopted by most clinical laboratories for the diagnosis of the disease. A recent survey found that only 3.6% of English National Health Service laboratories used the CCNA method for the detection of CDI (Goldenberg and French, 2011).

The RCCNA is a new commercially available method of cytotoxin testing, designed to test directly from stool and overcome the limitations of EIAs and previous CCNA testing methods. At the time of writing this report, no peer-reviewed literature could be found to have examined the validity of the RCCNA as a method of diagnosing toxigenic CDI.

We found the RCCNA method to have a high level of both sensitivity and specificity when compared to the EIA and TC. Against the commonly used EIA, RCCNA had a higher level of sensitivity (90.8% versus 78.6%) and similar specificity (98.3% versus 97.8%) as well as better scores for PPV (87.3% versus 81.9%) and NPV (98.8% to 97.3%), suggesting it is a more accurate method for CDI diagnosis. It must be noted that no significant difference could be found between the 2 test methods. The percentage of samples diagnosed by the RCCNA that were considered to be false negatives (9.2%) were lower than that by the EIA (21.4%) although both scores indicate an objectionable number of missed cases in a clinical setting. False-positive rates for both the RCCNA and EIA were much more acceptable at 1.7% and 2.2%, respectively. Unlike TC, RCCNA and EIA methods detect toxin levels from the stool and not from the culture. It has been argued therefore that TC detects the presence of toxigenic *C. difficile* strains in the patient but not whether they are pathogenic (Planche and Wilcox, 2011). This may have caused the high false-positive rates for both the RCCNA and EIA when compared to TC.

Previous studies using HFF cells have performed the test using microtitre trays and not the RCCNA. Stamper et al. (2009) recorded a sensitivity of 67.2% and a specificity of 99.1% compared to toxigenic culture. Reller et al. (2007) found the sensitivity rate of CCNA using HFF cells after a glutamate dehydrogenase (GDH) screen to be 77%. Another study has noted higher sensitivity levels. Aldeen et al. (2000) used HFF cells for CCNA recording a sensitivity of 97% and a specificity of 100% compared to a Toxin A/B ELISA. Other studies using Vero cells have recorded sensitivities and specificities of 86.4% and 99.2%, respectively (Eastwood et al., 2009). One study rated the sensitivity and specificity levels of Vero cells at 83.1% and 96.7%, respectively (Novak Weekley et al., 2010), although this was after both a GDH and an EIA screen.

CCNA is considered by some to be the GS for the detection of toxigenic CDI. RCCNA compared favorably to the reference method of toxigenic culture which is also advocated as a GS (Delmee et al., 2005). Other research comparing *C. difficile* diagnostic methods has reported much lower sensitivities for CCNA compared to TC (Tenover et al., 2010). Our reduced positive rates for TC could be due to the use of EIA as the toxin detection component of the algorithm (Carroll, 2011; She et al., 2009). The RCCNA does have the capability to be the toxigenic detection component of a TC algorithm, although it was only used with direct-from-stool samples in this study.

EIA alone appeared to miss approx. 20% of cases, with a sensitivity of approx. 80% compared to the other approaches. The specificity and NPV of the EIA were comparable and competitive with the other 2. She et al. (2009) compared the Premier Toxin A & B EIA to the CCNA method using HFF cells in microtitre wells and TC. They recorded higher sensitivity levels for the EIA (87.1%) than those recorded in this study but suggest that the EIA has a low reproducibility level and could give a high number of false-negative results. Planche et al. (2008) found the Meridian Premier EIA to have higher levels of sensitivity (95%) and specificity (97%). In their study, Planche et al. also highlighted the impact the prevalence of a disease can have on the PPV of a diagnostic method defining an acceptable test as one with a sensitivity of at least 90% and a false-positive rate of 3% or less. The RCCNA was therefore an acceptable method for detecting toxigenic CDI according to these guidelines. A difference of 5.4% in PPV between the RCCNA and the EIA is likely to be the result of the 1% difference in CDI prevalence recorded in our study.

CCNA was the cheapest of the 3 diagnostic methods with a cost of approximately £9.00 per sample. The EIA was slightly more expensive (~£11.00 per sample), although a result could be provided within an hour. RCCNA required up to 48 h for a confirmatory negative result; however, some positive cell lysing was observed from 2 to 6 h of incubation. Although seen by some as the GS and used as the reference method for this study, TC was the most expensive method at approx. £21.00 per sample. TC result turnaround time was also the longest at 96 h for a positive result.

The most common reason for repeating a specimens CCNA test was the phenomena of destruction to the cell monolayer or ‘bunching’, which occurred to both the specimen and control cell vials, suggesting it to be specimen specific and not handler error. Although 3.3% of samples were repeated for CCNA, this method still proved more cost-effective than using the EIA, which required no repeat testing. The reasons for the ‘bunching’ phenomena were unknown by the kit supplier or manufacturer and it was beyond the scope of this project to determine its cause, which increased the time to find a true result and processing costs.

Recent guidelines by the British Government's Department of Health advocate the use of a 2-step algorithm for the detection of toxigenic CDI and confirmed that EIA was not suitable as a standalone diagnostic test and should be preceded by a screening test of either GDH antigen EIA or nucleic acid amplification (Department of Health, 2012). This highlights the fact that CCNA is not considered a suitable method as part of a 2-step algorithm for the diagnosis of toxigenic *C. difficile*. Some laboratories have adopted polymerase chain reaction (PCR) within their testing algorithms for CDI, which aids the diagnosis of strain types and can highlight potentially hypervirulent strains such as PCR ribotype 027. A limitation to our study was that ribotyping of all positive *C. difficile* cultures to determine strain type was not carried out. A further limitation to the study was the inability to collect clinical details including antibiotic usage for all patients.

The Hs27 HFF ReadyCells are an easy-to-use and sensitive CCNA method for the detection of toxigenic *C. difficile* directly from stool. They do not require the technical expertise of cell line maintenance and are more cost-effective than other CCNA methods and some commonly used commercial laboratory tests. However, the turnaround times and ability to require repeat testing make it an unsuitable method to be used in a clinical laboratory setting. However, the RCCNA's ability to maintain GS sensitivities suggests that it would be a viable method in a nonclinical laboratory setting, such as a reference laboratory. Further research is required to understand the mechanisms that cause damage to the cell monolayer and increase the rate of repeat testing. Additionally, future studies of this algorithm would benefit from further clinical and diagnostic details such as antibiotic usage and *C. difficile* strain type.

## Figures and Tables

**Table 1:** Comparison of performance results for the RCCNA and EIA method compared to a toxigenic culture reference standard.

Test method^a^	No. of specimens		Performance (95% Confidence Interval)					
	Positive	Negative	Sensitivity (%)	Specificity (%)	PPV (%)	NPV (%)	False-positive rate (%)	False-negative rate (%)
TC	98	762						
EIA	94	766	78.6 (69.1–86.2)	97.8 (96.5–98.7)	81.9 (72.6–89.0)	97.3 (95.7–98.2)	2.2 (1.3–3.5)	21.4 (13.8–30.9)
RCCNA	102	758	90.8 (83.3–95.7)	98.3 (97.1–99.0)	87.3 (79.2–93.0)	98.8 (97.8–99.5)	1.7 (0.9–2.9)	9.2 (4.3–16.7)

a TC = Toxigenic culture; EIA = enzyme immunoassay; RCCNA = Hs27 ReadyCell cytotoxin cell neutralisation assay.
